# Impact of bronchiectasis on outcomes of hospitalized patients with acute exacerbation of chronic obstructive pulmonary disease: A propensity matched analysis

**DOI:** 10.1038/s41598-018-27680-y

**Published:** 2018-06-18

**Authors:** Ernesto Crisafulli, Mónica Guerrero, Antonella Ielpo, Adrian Ceccato, Arturo Huerta, Albert Gabarrús, Néstor Soler, Alfredo Chetta, Antoni Torres

**Affiliations:** 1 Department of Medicine and Surgery, Respiratory Disease and Lung Function Unit, University of Parma, Parma, Italy; 2 Pneumology Department, Clinic Institute of Thorax (ICT), Hospital Clinic of Barcelona - Institut d’Investigacions Biomèdiques August Pi i Sunyer (IDIBAPS) - University of Barcelona (UB), Barcelona, Spain

## Abstract

The coexistence of both Chronic Obstructive Pulmonary Disease (COPD) and bronchiectasis (BE) define an emerging phenotype with a worse prognosis; however, data about these patients do not consider baseline characteristics as confounders. We evaluate the impact of BE on outcomes of hospitalized patients with acute exacerbation of COPD (AECOPD). We prospectively considered AECOPD patients, analysed using a propensity score matching (PSM) method. The outcomes included length of hospital stay, use of non-invasive and invasive mechanical ventilation, intensive care unit admission, and mortality up to 3-years. Out of the 449 patients enrolled, 160 had associated BE. AECOPD with BE were older, had lower body mass index and greater functional impairment and severity of symptoms than AECOPD without BE. After PSM, 91 patients were considered for each group and no significant differences were found for all baseline characteristics. In full cohort, the cumulative mortality rate, the survival time, the Kaplan-Meier survival curves and the risk of death were worse in AECOPD with BE in the follow-up of 6-months, 1-year and 3-years. After PSM, data on mortality were similar between AECOPD with and without BE. In conclusion, in AECOPD patients the presence of BE does not influence mortality in a long-term follow-up.

## Introduction

Chronic Obstructive Pulmonary Disease (COPD) is a non-communicable disease representing the third cause of death worldwide^1^. During the natural course of COPD many patients experience acute exacerbation (AECOPD) characterized by a deterioration of respiratory signs and symptoms^2^ and an increase of inflammatory response^3^. Bronchiectasis (BE) is a chronic respiratory condition related to a dilatation of bronchi and airway wall thickening on imaging of chest computed tomography (CT) scan^4^; this irreversible alteration may lead to recurrent episodes of bronchial infections, inflammation, airway obstruction and progressive lung destruction^5^.

In order to define the risk stratification of COPD patients^6^, specific risk factors (age^7^, smoking^8^, sex^9^), clinical phenotypes (frequent exacerbators^10^, low body mass index-BMI^11^, increased dyspnoea^12^), and measurements of disease severity (forced expiratory volume in the 1^st^ second-FEV_1_^13^) are associated to worse prognosis. For this reason, these measured baseline characteristics (covariates) may be considered as predictors of mortality^7–13^.

At present, COPD is not considered a cause of BE and patients who fulfil both diagnostic criteria may be identified in an overlap condition^14^ having a prevalence between 27% and 69%^4,15–18^. Although the presence of BE has been reported as an unfavourable feature for COPD^15,17,19–21^, the role of the baseline covariates^7–13^ that are different between COPD patients with and without BE^16,17,22^ have been not entirely evaluated. Although the mortality risk of BE in COPD patients has been evaluated with regression adjustment^15,17,18,20^, residual confounding factors by unmeasured or inadequately measured baseline covariates may account for the rest of the risk. Comparability between groups should be a requirement in observational studies, avoiding indication bias, a specific type of selection bias^23^; the use of restriction may minimize bias^23,24^. Propensity score (PS)^25^ represents the estimated probability of exposure assignment conditional on observed baseline covariates. Propensity score matching (PSM) matches patients in each group based on the similarity of their PS and the distribution of observed baseline covariates will be similar between exposed and unexposed subjects, reducing the effect of confounding variables^26^.

Our study hypothesis was that potential baseline covariates may influence the clinical impact and prognosis of hospitalized AECOPD patients with BE in a short and long-term follow-up. Using a PSM method and eliminating baseline differences between AECOPD patients with and without BE, we could evaluate the impact of BE that is still lacking in AECOPD patients.

## Methods

### Study Design

This was a prospective study conducted at the Hospital Clinic of Barcelona (Spain) in a period of 7 years between May 2009 and May 2016. The sampling method was systematic and all AECOPD patients admitted to our Pneumology Department were enrolled in the study.

### Patients Selection

The patients included had to meet COPD criteria according to the GOLD document^27^. Spirometry was performed in the stable phase and at least six months prior admission to hospital and a smoking history of 20 pack/years was considered as a positive habit. Definition of AECOPD was based on worsening of respiratory symptoms compared with preceding days requiring a change in domiciliary therapy^27^; the hospitalization was based on the severity of AECOPD according to the respiratory signs or symptoms and the presence of potential indicators^27^.

The presence of BE was detected by a chest CT scan, performed during hospitalization or in a period of at least six months before hospital admission. The radiological features of BE collected regards type (cylindrical, cystic or both), distribution (upper, middle or lower lobes or associated lobes), position (lung right, left or bilateral), and extension (or ≥3 involved lobes). Patients without a chest CT scan available were classified as AECOPD without BE.

### Exclusion Criteria

The exclusion criteria concerned patients with a documented history of other concomitant chronic respiratory disease (asthma, cystic fibrosis) and patients in whom a community-acquired pneumonia or an acute heart failure were identified clinically and by chest X-ray or CT scan at admission.

### Ethics statement

The Hospital’s Ethics Committee approved the study protocol (CEIC 2008/4106), conducted according to the Good Clinical Practices and the declarations of Helsinki. An informed consent have been obtained from all enrolled patients.

### Microbiological Sample collection

On the first day of hospitalization sputum sample was collected from spontaneous cough; if the sample was adequate (a count of more than 25 leukocytes and less than 10 epithelial cells per field) it was processed using Gram stain and sputum culture. In patients without a spontaneous sputum sample an induced sputum production was obtained by an inhalation of a 5% hypertonic saline solution for 5 to 10 minutes delivered via a nebulizer device.

### Measurements

Data about demographic variables, body mass index (BMI), smoking habit (current or former) with number of pack/year, number of comorbidities (Charlson index), prevalence of ischemic heart disease and diabetes, dyspnea grade measured by the modified Medical Research Council (mMRC) scale, severity of disease (COPD severity score measured by a COPD-SS questionnaire), and use of long-term oxygen therapy (LTOT) were recorded. Season of occurrence of AECOPD, characteristics and number of previous AECOPD occurring in the preceding year and data on home care medications (inhaled bronchodilators as short-acting β_2_ agonist [SABA], long-acting β_2_ agonist [LABA], anticholinergics, inhaled steroids [ICS]) were also recorded.

Vital signs (body temperature, respiratory and heart rate, systolic and diastolic blood pressure) were assessed at admission. At admission and at day 3 we recorded data about gas analysis (pH, partial arterial carbon dioxide pressure [PaCO_2_], the ratio of partial arterial oxygen pressure to the fraction of inspired oxygen [PaO_2_/FiO_2_], serum bicarbonate [HCO_3_^−^], and base excess [BE]), systemic response (leukocytes, haematocrit, haemoglobin, C-reactive protein [CRP], glucose, and creatinine). Data on number of patients using systemic corticosteroids and/or antibiotics, duration of antibiotic treatment and classes of antibiotics used were also recorded.

### Outcomes

Length of hospital stay (LOS), use of non-invasive and invasive mechanical ventilation (NIMV and IMV), and intensive care unit (ICU) admission were considered as variables of clinical progression. Data on prognosis (cumulative number of deaths for all-causes, estimated time to death) were recorded in a follow-up of 30 days, 6 months, 1 year and 3 years. The date of death was identified by centralized registries.

### Statistical analysis

A total sample size of 182 patients (91 patients in the group of AECOPD with BE and 91 patients in the group of AECOPD without BE, according to 1:1 allocation ratio) was estimated to provide at least a 80% power and a two-sided alpha value of 0.05 to detect as statistically significant an absolute difference of 15% in the percentage of 3-years mortality of patients between groups (20% patients with BE *vs* 5% patients without BE)^17^.

Data were reported with number and percentage of patients for categorical variables, means ± standard deviation (SD) or medians [1^st^ quartile; 3^rd^ quartile] for continuous variables with normal and non-normal distribution, respectively. Categorical variables were compared using the *X*^2^ test or the Fisher exact test while continuous variables with the *t* test or the non-parametric Mann-Whitney test.

**Figure 1:**
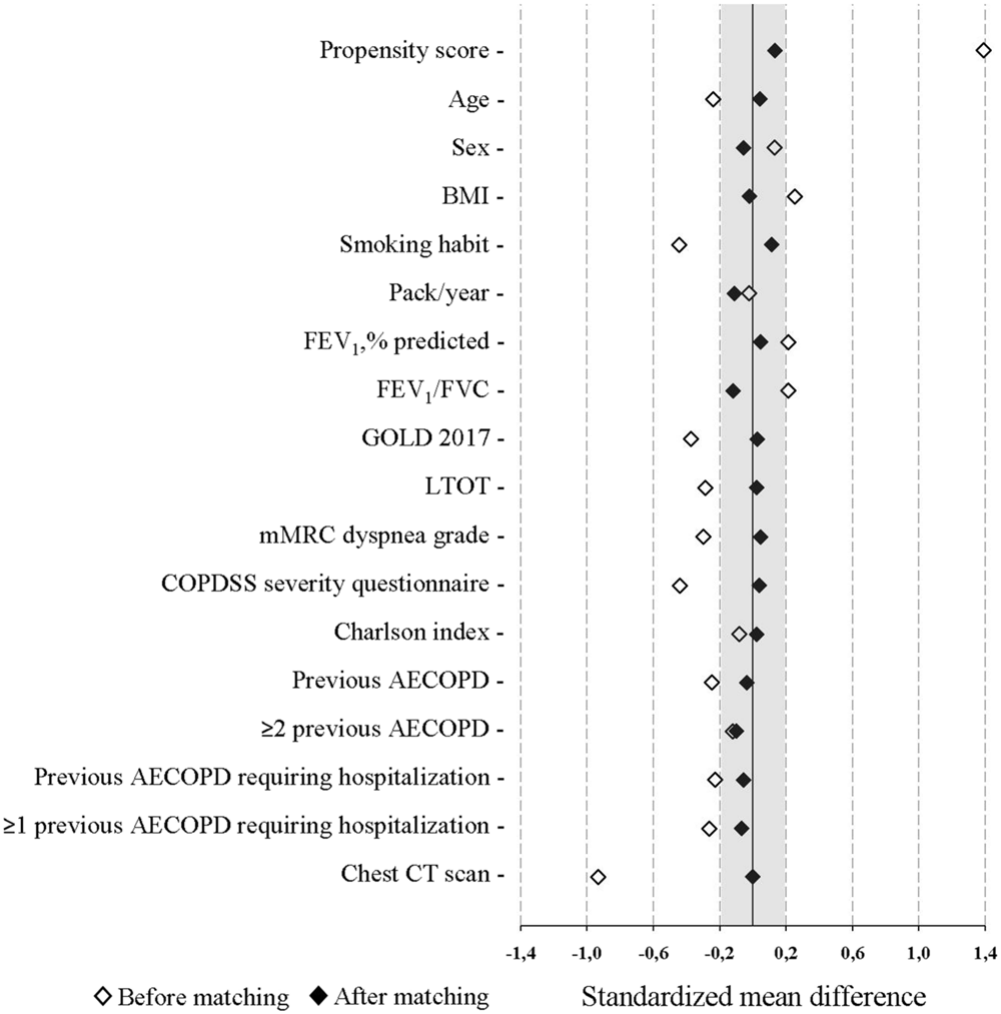
Plot displaying standardized mean differences in baseline characteristics between AECOPD patients without and with BE, before and after propensity score matching. *Abbreviations:* BE indicates bronchiectasis; BMI, body mass index; FEV_1_, forced expiratory volume in the 1^st^ second; FVC, forced vital capacity; LTOT, long-term oxygen therapy; mMRC, modified Medical Research Council; COPD-SS, COPD severity score questionnaire; AECOPD acute exacerbation of COPD.

PS was used to obtain the balance among baseline variables between AECOPD patients with and without BE listed in Table 1. A PSM program^28^ was used to match the two cohorts using a 1:1 nearest neighbour matching, without replacement within a caliper width of 0.2. Variables were chosen for inclusion in the PS calculation according to the methods of Brookhart *et al*.^29^ and included variables associated with hospitalized AECOPD patients with BE and outcome (age, BMI, FEV_1_, smoking habit, COPD-SS questionnaire, chest CT scan, and patients with ≥2 previous AECOPD). After matching, an adequate comparability was shown by a decrease to <20% (0.2) of the standardized mean difference^30^ between AECOPD with and without BE for all baseline covariates (Fig. 1); moreover, an adequate model fit with discrimination and calibration of the PS was demonstrated by the logistic model including covariates yielded a Goodness-of-Fit p = 0.321.

**Table 1:** Baseline characteristics of AECOPD patients evaluated in the full cohort and in the propensity score matching sample.

Variables	AECOPD (Full cohort)(n = 449)			AECOPD (Propensity score matching)*(n = 182)		
	Without BE(n = 289)	With BE(n = 160)	p-value	Without BE(n = 91)	With BE(n = 91)	p-value
Age, years	70.4 ± 10.2	72.7 ± 8.6	**0.014**	70.9 ± 9.4	71.3 ± 8.6	0.761
Male, %	79	84	0.183	82	85	0.689
BMI, kg/m^2^	27.8 ± 5.6	26.5 ± 4.9	**0.022**	27.4 ± 5.0	27.3 ± 5.1	0.887
Smoking habit: Current/Former,%	47/53	26/74	**<0.001**	35/65	30/70	0.428
Pack/year	60 [40; 80]	59.5 [40; 80]	0.917	60 [40; 80]	50 [40; 80]	0.430
FEV_1_,% predicted	48.5 ± 18.9	44.6 ± 17.2	**0.040**	46.2 ± 17.8	47.0 ± 17.7	0.758
FEV_1_/FVC	51.2 ± 14.6	48.0 ± 15.1	**0.047**	50.7 ± 14.2	48.9 ± 16.6	0.446
GOLD 2017 stages: A/B/C/D,%	31/37/12/21	17/33/16/33	**0.017**	22/36/19/22	21/36/18/24	0.991
LTOT, %	23	36	**0.004**	23	24	0.861
mMRC dyspnea grade	2 [1; 3]	2 [1; 3]	**0.014**	2 [1; 3]	2 [1; 3]	0.670
COPD-SS severity questionnaire	12 [7; 17.5]	16 [11; 20]	**<0.001**	14 [9; 17]	14 [9; 18]	0.798
Charlson index	2 [1; 3]	2 [1; 3]	0.174	2 [1; 3]	2 [1; 4]	0.785
Ischemic heart disease, %	9	11	0.582	8	10	0.601
Diabetes, %	23	22	0.866	19	20	0.851
Season of admission:Winter/Spring/Summer/Autumn, %	44/14/26/17	41/17/28/13	0.543	41/19/27/13	37/18/31/14	0.947
Previous AECOPD^†^	0 [0; 1]	1 [0; 2]	**0.042**	1 [0; 2]	0 [0; 1]	0.343
Patients with ≥2 previous AECOPD^†^, %	25	30	0.212	27	23	0.495
Previous AECOPD requiring hospitalization^†^	0 [0; 1]	0 [0; 1]	**0.006**	0 [0; 1]	0 [0; 1]	0.717
Patients with ≥1 previous AECOPD requiring hospitalization^†^, %	29	41	**0.007**	34	31	0.635
Patients having a chest CT scan#, %	73	100	**<0.001**	100	100	>0.999
Salbutamol only, %	5	1	0.090	2	0	0.497
Anticholinergic only, %	6	5	0.609	3	8	0.206
LABA+Anticholinergic, %	2	1	0.412	2	1	>0.999
LABA+ICS, %	3	2	0.747	2	4	0.678
Anticholinergic+ICS, %	1	3	0.437	1	1	>0.999
LABA+Anticholinergic+ICS, %	36	38	0.702	41	38	0.656

Data are shown as number of patients (percentage), means ± standard deviation or medians [1^st^ quartile; 3^rd^ quartile], unless otherwise stated. Percentages are calculated on non-missing data.

*Abbreviations:* AECOPD indicates acute exacerbation of COPD; BE, bronchiectasis; BMI, body mass index; FEV_1_, forced expiratory volume in the 1^st^ second; FVC, forced vital capacity; GOLD, global initiative for chronic obstructive lung disease; LTOT, long-term oxygen therapy; mMRC, modified Medical Research Council; COPD-SS, COPD severity score questionnaire; LABA, long-acting β_2_ agonist; ICS, inhaled corticosteroids.

LABA includes salmeterol, formoterol and indacaterol; Anticholinergic includes ipratropium and tiotropium; and ICS includes budesonide and fluticasone.

*The variables included as covariates in the propensity score matching were: age, body mass index, forced expiratory volume in the 1^st^ second; smoking habit, COPD-SS severity questionnaire, chest CT scan, and patients with ≥2 previous AECOPD.

^†^Previous AECOPD were considered if occurring in a period of the preceding year.

^#^The chest CT scan was obtained in a period of six months prior the hospitalization or during the hospitalization.

Time-to event variables were analysed by means of Kaplan-Meier survival curves and a Gehan-Breslow-Wilcoxon test was applied because this test emphasizes early differences^31^. Patients lost to follow-up were censored in the survival analysis. Cox proportional hazard regression models were used in mortality at 30-days, 6-months, 1-year, and 3-years^32^. The hazard ratio (HR) and its 95% confidence intervals (CI) were calculated.

All statistical analyses were performed using IBM SPSS Statistics 24.0 (Armonk, New York, USA). A value of p < 0.05 was considered statistically significant.

### Data availability

The datasets generated during and/or analysed during the current study are available from the corresponding author on reasonable request.

## Results

### Baseline characteristics

**Figure 2:**
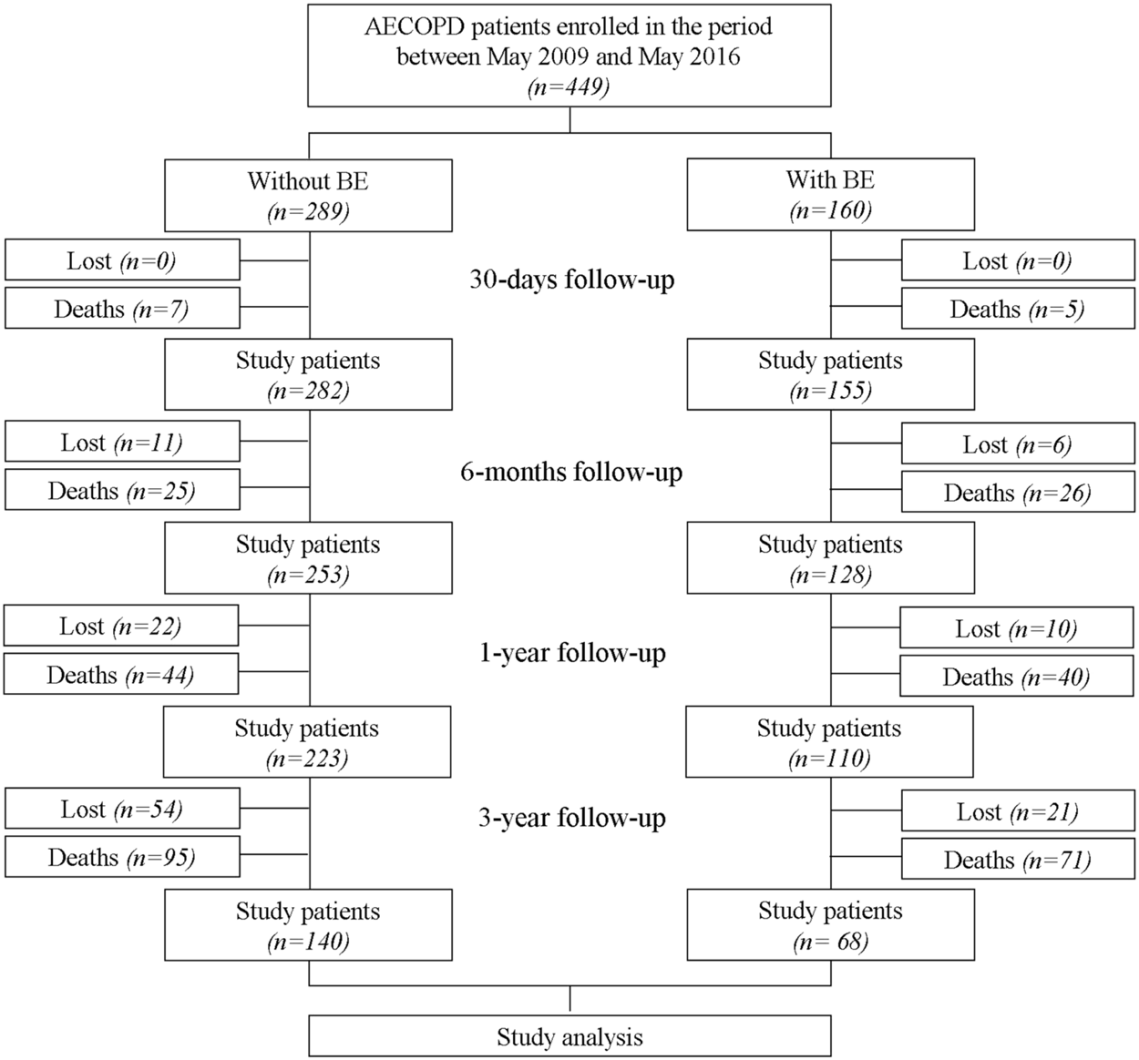
Study flow chart. *Abbreviations:* AECOPD stands for acute exacerbation of COPD; BE, bronchiectasis.

449 consecutive AECOPD patients (81% men) with a mean age of 72 years were considered; of these, 160 patients (36%) had associated BE. The chest CT scan was available in 330 patients (73%); in the PSM sample only patients having a chest CT scan (n = 182) were considered. Figure 2 shows the study flow diagram.

In full cohort, AECOPD patients with BE were older, with a lower BMI and greater functional impairment, severity of symptoms and questionnaire-reported severity characteristics than AECOPD patients without BE. Moreover, patients with BE were more frequently former smokers, with need for LTOT and had a significant history of AECOPD in the previous year, also requiring hospitalization. After PSM, no significant differences were found in all baseline characteristics (see Table 1).

**Figure 3:**
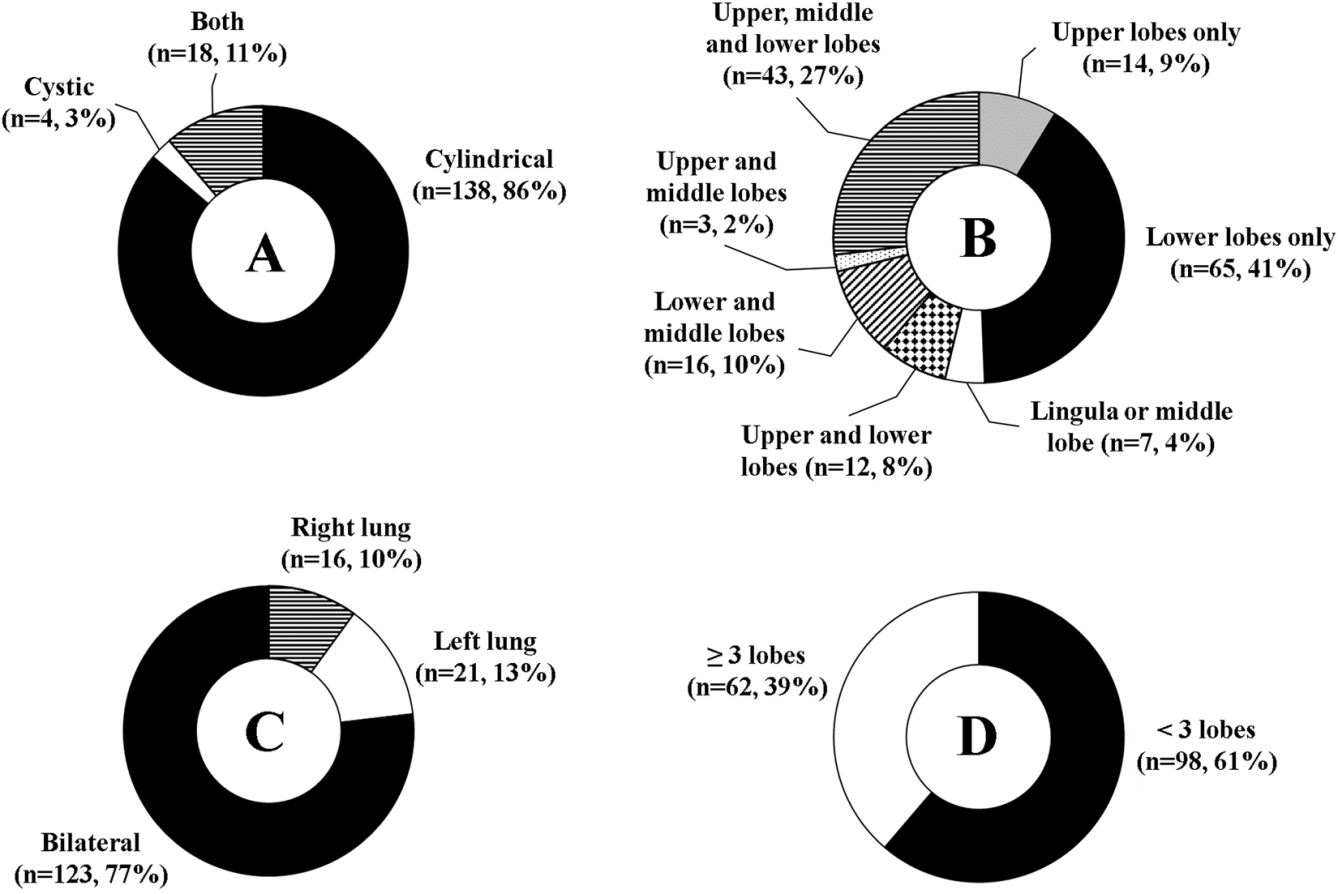
Radiological features of AECOPD patients with BE. Circles **A**,**B**,**C**, and **D** show the number and percentage of patients according to the radiological features (type, distribution, position, and extension, respectively).

Concerning the radiological aspects of BE, the cylindrical type (circle A) with a distribution in the lower lobes (circle B), in a bilateral position (circle C) and involving ≤3 lobes (circle D) represent the most prevalent features (86%, 41%, 77%, and 61%, respectively) (Fig. 3).

### Clinical, laboratory and microbiological variables

With regard to clinical and laboratory variables (Table 2), AECOPD patients with BE in comparison to patients without BE showed a higher C-reactive protein (CRP) level at day 3 (median 1.7 mg/dL *vs* 0.9 mg/dL, p = 0.026) in full cohort. In the PSM cohort, at admission lower levels of PaCO_2_ (42.1 mmHg *vs* 49.1 mmHg, p = 0.003), HCO_3_^−^ (26 mmol/L *vs* 28 mmol/L, p = 0.001), BE (1.4 mmol/L *vs* 2.7 mmol/L, p = 0.015) and glucose (117 mg/dL *vs* 129 mg/dL, p = 0.028) were shown in AECOPD patients with BE. The other clinical and laboratory variables presented similar values in the two groups in both full and PSM cohort.

**Table 2:** Clinical and laboratory variables recorded at admission and at day 3.

Variables	AECOPD (Full cohort)			AECOPD (Propensity score matching)		
	Without BE	With BE	p-value	Without BE	With BE	p-value
Respiratory rate at admission, b/min	24 [20; 28]	24 [20; 28]	0.716	23 [20; 26]	24 [20; 28]	0.544
Heart rate at admission, b/min	92 [81; 104]	94 [84; 108]	0.199	90 [81; 100]	91 [83; 108]	0.329
Body temperature at admission, °C	36.4 [36; 36.9]	36.3 [35.9; 36.9]	0.760	36.2 [35.9; 36.7]	36.3 [35.7; 36.9]	0.841
SBP at admission, mmHg	138 [120; 156]	139 [124; 155]	0.531	137 [119; 152]	143 [122; 157]	0.156
DBP at admission, mmHg	75.5 [67; 87]	76 [69; 86]	0.780	76 [68; 86]	79 [69; 87]	0.574
pH at admission	7.39 [7.33; 7.43]	7.39 [7.36; 7.43]	0.112	7.39 [7.33; 7.44]	7.41 [7.37; 7.44]	0.081
*- at day 3*	7.41[7.39; 7.44]	7.40 [7.37; 7.43]	0.061	7.41 [7.38; 7.45]	7.40 [7.36; 7.43]	0.334
PaCO_2_ at admission, mmHg	48.8 [39.4; 61.4]	45.9 [37.4; 56.5]	0.136	49.1 [40.4; 57.9]	42.1 [36.7; 50.8]	**0.003**
*- at day 3*	46.6 [41.2; 53.5]	49.5 [41.7; 55.9]	0.461	46 [39; 54.4]	48.5 [42.3; 53.3]	0.582
PaO_2_/FiO_2_ at admission, mmHg	262 [215; 318]	259 [225; 314]	0.855	260 [206; 315]	259 [228; 334]	0.476
*- at day 3*	286 [252; 316]	279 [253; 314]	0.885	287 [250; 331]	279 [250; 319]	0.601
HCO_3_ at admission, mmol/L	28 [25; 33]	28 [24; 31]	0.112	28 [26; 32]	26 [23; 29]	**0.001**
*- at day 3*	30 [26; 33]	30 [27; 32]	0.746	31 [26; 35]	28 [27; 30]	0.344
BE at admission, mmol/L	2.4 [0.1; 6]	2.2 [−0.4; 5.2]	0.400	2.7 [0.6; 5.9]	1.4 [−0.8; 3.7]	**0.015**
*- at day 3*	4.4 [2.0; 7.4]	4.2 [1.5; 6.7]	0.808	4.4 [0.1; 7.4]	3.4 [1.3; 5.8]	0.880
Leucocytes at admission, x 10^9^/l	10.1 [7.7; 13.8]	10.2 [8; 14]	0.586	10.1 [7.3; 13.8]	10.7 [8.5; 14.1]	0.167
*- at day 3*	10.9 [8.3; 12.9]	10.5 [8.2; 13.5]	0.800	10.6 [7.9; 12.3]	10.3 [8.5; 13.4]	0.365
Haematocrit at admission,%	43 [40; 47]	43 [39; 47]	0.445	43 [39; 47]	44 [40; 48]	0.330
*- at day 3*	41 [37; 45]	40 [37; 44]	0.330	41 [37; 44]	42 [37; 45]	0.916
Haemoglobin at admission, g/L	140 [127; 152]	137 [124; 151]	0.339	138 [126; 151]	143 [126; 154]	0.319
*- at day 3*	132 [119; 143]	129 [115; 143]	0.270	134 [118; 144]	136 [118; 146]	>0.999
C-reactive protein at admission, mg/dL	3.7 [1.1; 9.5]	3.7 [1.5; 10.5]	0.316	4.0 [1.4; 9.4]	3.2 [1.5; 7.6]	0.764
*- at day 3*	0.9 [0.3; 2.9]	1.7 [0.4; 4.7]	**0.026**	0.9 [0.4; 3.1]	1.6 [0.3; 4.2]	0.330
Glucose at admission, mg/dL	126 [109; 161]	122 [104; 159]	0.192	129 [113; 165]	117 [103; 150]	**0.028**
*- at day 3*	122 [98; 159]	119 [96; 151]	**0.426**	127 [101; 155]	116 [95; 149]	0.337
Creatinine at admission, mg/dL	0.9 [0.8; 1.1]	0.9 [0.8; 1.1]	0.278	0.9 [0.8; 1.1]	1.0 [0.8; 1.1]	0.968
*- at day 3*	0.8 [0.7; 1.1]	0.9 [0.8; 1.1]	0.056	0.9 [0.7; 1.2]	0.9 [0.8; 1.1]	0.329
Patients using systemic corticosteroids, %	90	94	0.211	89	93	0.284
Patients using antibiotics, %	85	89	0.155	87	94	0.115
Duration of antibiotic treatment, days	7 [5.5; 10]	7 [5; 10]	0.791	7 [5; 9]	7 [5; 10]	0.383
Penicillins, %	18	16	0.779	9	14	0.293
Fluoroquinolones, %	50	52	0.731	48	56	0.333
Macrolides, %	2	1	>0.999	0	1	>0.999
Cefalosporins, %	5	1	0.144	4	0	0.103
Carbapenems, %	1	0	0.532	1	0	0.473

Data are shown as number of patients (percentage), means ± standard deviation or medians [1^st^ quartile; 3^rd^ quartile], unless otherwise stated. Percentages are calculated on non-missing data.

*Abbreviations:* AECOPD indicates acute exacerbation of COPD; BE, bronchiectasis; SBP and DBP, systolic and diastolic blood pressure, respectively; PaCO_2_, partial arterial carbon dioxide pressure; PaO_2_/FiO_2_, ratio of partial arterial oxygen pressure to the fraction of inspired oxygen; HCO_3_^−^, serum bicarbonate; BE, base excess.

Systemic corticosteroids include methylprednisolone; Penicillins includes amoxicillin and amoxicillin/clavulanate; Fluoroquinolones includes ciprofloxacin, moxifloxacin, and levofloxacin; Macrolides includes azithromycin and clarithromycin; Cefalosporins includes ceftriaxone, cefotaxime, cefuroxime and cefepime; and Carbapenems includes meropenem.

In full cohort, a greater prevalence of *Pseudomonas aeruginosa* (38% *vs* 19%, p = 0.037) and a lower prevalence of *Haemophilus influenzae* (8% *vs* 24%, p = 0.049) were shown in AECOPD patients with BE in comparison to patients without BE; after matching, all microbiological variables were similar between groups (Table 3).

**Table 3:** Microbiological variables.

	AECOPD (Full cohort)			AECOPD (Propensity score matching)		
	Without BE	With BE	p-value	Without BE	With BE	p-value
Patients with positive cultures in sputum*	53 (18)	39 (24)	0.150	18 (20)	23 (25)	0.442
*Pseudomonas aeruginosa*	10 (19)	15 (38)	**0.037**	5 (28)	7 (30)	0.853
*Haemophilus influenzae*	13 (24)	3 (8)	**0.049**	5 (28)	1 (4)	0.070
*Streptococcus pneumoniae*	10 (19)	8 (20)	0.844	3 (17)	7 (30)	0.467
*Staphylococcus spp*	5 (9)	2 (5)	0.695	2 (11)	1 (4)	0.573
*Pasteurella*	0 (0)	2 (5)	0.177	0 (0)	1 (4)	>0.999
*Moraxella catarrhalis*	3 (6)	0 (0)	0.259	0 (0)	0 (0)	—
*Candida spp*	1 (2)	1 (3)	>0.999	0 (0)	1 (4)	>0.999
*Aspergillus*	0 (0)	2 (5)	0.177	0 (0)	1 (4)	>0.999
*Serratia*	1 (2)	0 (0)	>0.999	0 (0)	0 (0)	—
*Mycobacterium no-TBC*	0 (0)	1 (3)	0.424	0 (0)	1 (4)	>0.999
*Polymicrobial etiology*	10 (18)	5 (13)	0.508	3 (17)	3 (13)	>0.999
Virus-positive patients*	15 (5)	7 (4)	0.714	5 (5)	4 (4)	>0.999
*Influenza B virus*	1 (7)	1 (14)	>0.999	0 (0)	0 (0)	—
*Respiratory syncytial virus*	6 (40)	1 (14)	0.430	1 (20)	0 (0)	>0.999
*Rhinovirus*	4 (27)	3 (43)	0.704	1 (20)	2 (50)	>0.999
*Parainfluenza virus type 1*	2 (13)	1 (14)	>0.999	1 (20)	1 (25)	>0.999
*Parainfluenza virus type 3*	2 (13)	0 (0)	0.540	2 (40)	0 (0)	0.497
*Parainfluenza virus type 4*	0 (0)	1 (14)	0.356	0 (0)	1 (25)	>0.999

Data are shown as number of patients (percentage).

*The percentages are related to the number of patients without and with BE.

The percentages of pathogens are related in each group to the number of patients with positive cultures in sputum or a virus-positivity.

*Abbreviations:* AECOPD indicates acute exacerbation of COPD; BE, bronchiectasis.

### Outcomes

In the full cohort, AECOPD with BE in comparison to patients without BE showed a lower prevalence of NIMV (15% *vs* 25%, p = 0.011) and ICU admission (7% *vs* 15%, p = 0.016); the PSM cohort confirm data about lower prevalence of ICU admission in AECOPD with BE (6% *vs* 14%, p = 0.047) (Table 4).

**Table 4:** Study outcomes.

Variables	AECOPD (Full cohort)			AECOPD (Propensity score matching)		
	Without BE	With BE	p-value	Without BE	With BE	p-value
LOS, days	8 [6; 11]	8 [6; 10]	0.864	8 [6; 12]	7 [6; 10]	0.290
NIMV, %	25	15	**0.011**	23	13	0.077
IMV, %	4	4	0.830	2	3	>0.999
ICU admission, %	15	7	**0.016**	14	6	**0.047**
30-days mortality, n (%)	7 (2)	5 (3)	0.762	0 (0)	1 (1)	>0.999
Survival time	29.5[29.2 to 29.9]	29.7[29.3 to 30.0]	0.668	—	—	—
6-months mortality, n (%)	25 (9)	26 (17)	**0.015**	4 (5)	4 (5)	>0.999
Survival time	170.5 [166.6 to 174.4]	163.9 [157.6 to 170.3]	**0.016**	176.8 [173.3 to 180.3]	175.3 [170.6 to 179.9]	0.991
1-year mortality, n (%)	44 (16)	40 (27)	**0.013**	8 (10)	10 (12)	0.781
Survival time	328.1 [318.2 to 338]	304.2 [287.6 to 320.8]	**0.009**	343.4 [331.4 to 355.4]	339.0 [325.3 to 352.8]	0.688
3-years mortality, n (%)	95 (40)	71 (51)	**0.045**	26 (36)	26 (34)	0.764
Survival time	852.1 [810.3 to 894]	766.8 [701.9 to 831.8]	**0.014**	909.0 [844.1 to 974.0]	919.9 [853.7 to 986.1]	0.762

Data are shown as number of patients (percentage) or medians [1^st^ quartile; 3^rd^ quartile], unless otherwise stated. Percentages are calculated on non-missing data. Data for mortality was reported as cumulative. Data for survival time was calculated as mean [95% confidence interval] and reported as days.

*Abbreviations:* AECOPD indicates acute exacerbation of COPD; BE, bronchiectasis; LOS, length of stay in hospital; NIMV and IMV, noninvasive and invasive mechanical ventilation, respectively; ICU, intensive care unit.

In the propensity score matching group the survival time for 30-days mortality was not computed because all cases are censored. There is only one valid survival function value per group in at least one stratum.

**Figure 4:**
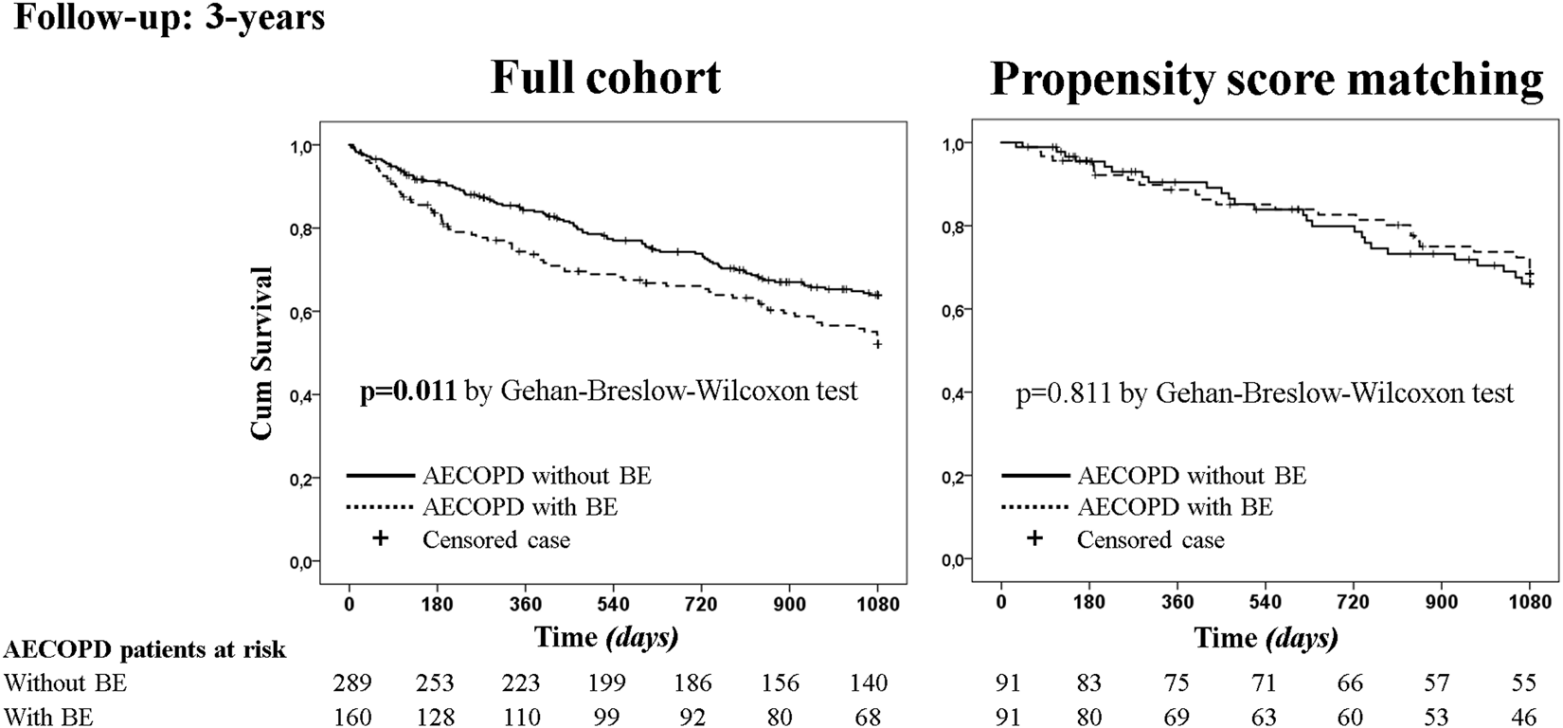
Kaplan-Meier survival curves in the follow-up period of 3-years in full cohort and in the propensity score matching cohort. *Abbreviations:* AECOPD stands for acute exacerbation of COPD; BE, bronchiectasis.

In full cohort, the cumulative mortality rate was significantly higher in AECOPD patients with BE in comparison to patients without BE in the follow-up of 6-months, 1-year and 3-years (17% *vs* 9%, p = 0.015; 27% *vs* 16%, p = 0.013; 51% *vs* 40%, p = 0.045; respectively). Moreover, the mean survival time was lower in AECOPD patients with BE (mean 163.9 days *vs* 170.5 days, p = 0.016 at 6-months; 304.2 days *vs* 328.1 days, p = 0.009 at 1-year; 766.8 days *vs* 852.1 days, p = 0.014 at 3-years) (Table 4). The Kaplan-Meier survival curves (Fig. 4) showed an unfavourable role of AECOPD patients with BE (Gehan-Breslow-Wilcoxon test p = 0.011 in the follow-up of 3 years). In the follow-up of 6-months, 1-year and 3-years, Cox regression (Table 5) showed an increased risk of death for all-causes in AECOPD patients with BE (HR [95% CI] 1.94 [1.12 to 3.36], p = 0.018; 1.73 [1.13 to 2.65], p = 0.012; 1.39 [1.02 to 1.89], p = 0.036; respectively). After PSM, the mortality rate, the mean survival time, the Kaplan-Meier survival curves, and the risk of death were similar between AECOPD patients with and without BE (Table 4, Fig. 4 and Table 5, respectively).

**Table 5:** Cox regression models evaluating the risk of all-causes death for AECOPD with BE.

	HR	95% CI	p-value
30-days mortality			
Crude (full cohort)	1.28	0.40 to 4.04	0.669
Propensity score matching	—	—	—
6-months mortality			
Crude (full cohort)	1.94	1.12 to 3.36	**0.018**
Propensity score matching	0.97	0.24 to 3.91	0.977
1-year mortality			
Crude (full cohort)	1.73	1.13 to 2.65	**0.012**
Propensity score matching	1.14	0.45 to 2.90	0.771
3-years mortality			
Crude (full cohort)	1.39	1.02 to 1.89	**0.036**
Propensity score matching	0.91	0.53 to 1.58	0.760

*Abbreviations:* AECOPD indicates acute exacerbation of COPD; BE, bronchiectasis; HR, hazard ratio; CI, confidence interval.

Cox regression for 30-days mortality in the propensity score matching sample cannot compute because all cases are censored.

**Figure 5:**
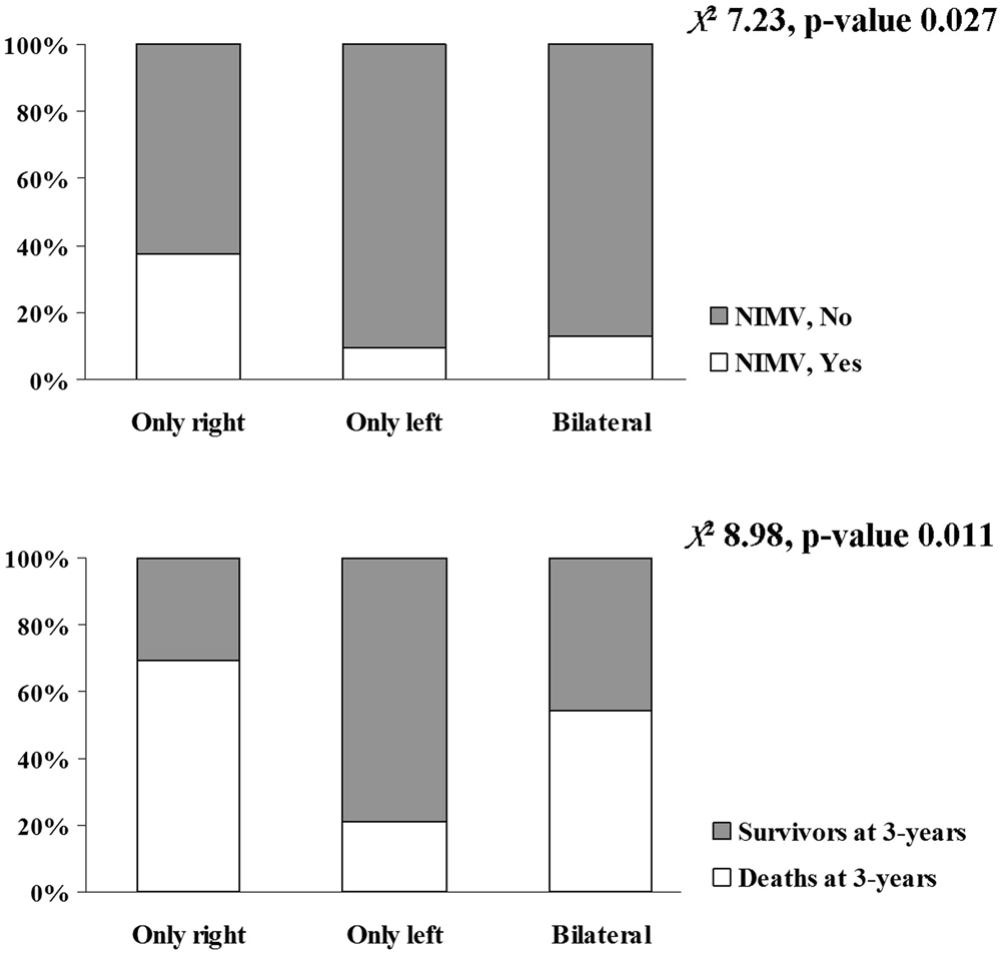
Prevalence of radiological features of AECOPD patients with BE according to the outcomes. *Abbreviations:* NIMV stands for non-invasive mechanical ventilation.

Concerning the prevalence of radiological features of BE according to the outcomes (Fig. 5), the only right position in comparison to only left and bilateral was respectively associated to a higher prevalence of NIMV (37%, 9% and 13%, p = 0.027) and survivors at 3-years (69%, 21% and 54%, p = 0.011). Data about type, distribution, and extension of BE had not influenced all outcomes.

In the comparison of baseline covariates between survivors and deaths (Table 6), in full cohort, in the follow-up of 6-months, 1-year and 3-years, deaths were among significantly older patients, former smokers, patients with greater staging severity, needing LTOT, with a higher dyspnoea grade, a greater questionnaire-reported severity and a higher number of previous AECOPD also needing hospitalization. BMI, FEV_1_% predicted and Charlson index were respectively worse in deaths at the follow-up of 1-year and 3-years, while prevalence of male gender were higher at 3-year follow-up only. In the PSM cohort, the Charlson index was different among survivors and death in the follow-up of 1-year and 3-years, while age, GOLD 2017 stage, LTOT, mMRC, and COPD-SS questionnaire were different in the follow-up of 3-years.

**Table 6:** Comparison of baseline covariates between survivors and deaths, in the full cohort and in the propensity score matching sample, in the follow-up of 6-months, 1-year and 3-years.

Variables	6-months			1-year			3-years		
	Survivors	Deaths	p-value	Survivors	Deaths	p-value	Survivors	Deaths	p-value
Age, years	70.6 ± 9.8	76.9 ± 7.3	**<0.001**	70.5 ± 9.7	75.2 ± 8.6	**<0.001**	69.4 ± 10.1	75.0 ± 8.2	<**0.001**
*Propensity score matching*	70.9 ± 9.0	76.8 ± 7.2	0.067	71.3 ± 8.8	71.8 ± 9.2	0.831	70.3 ± 9.4	73.7 ± 7.4	**0.018**
Male, %	81	90	0.120	82	89	0.096	80	92	**0.002**
*Propensity score matching*	85	75	0.350	86	89	>0.999	86	92	0.229
BMI, kg/m^2^	27.4 ± 5.5	26.6 ± 4.2	0.451	27.7 ± 5.4	25.6 ± 4.7	**0.009**	28.2 ± 5.6	25.9 ± 4.6	<**0.001**
*Propensity score matching*	27.3 ± 5.1	28.4 ± 4.1	0.535	27.4 ± 5.1	27.6 ± 4.4	0.878	27.8 ± 5.4	26.9 ± 4.3	0.315
Smoking habit: Current/Former, %	42/58	16/84	**<0.001**	44/56	19/81	**<0.001**	46/54	22/78	<**0.001**
*Propensity score matching*	33/67	13/87	0.439	31/69	33/67	0.858	34/66	23/77	0.165
FEV_1_,% predicted	47.4 ± 18.6	42.9 ± 16.8	0.150	47.8 ± 18.2	42.7 ± 17.4	**0.034**	48.9 ± 17.8	41.8 ± 16.1	<**0.001**
*Propensity score matching*	46.3 ± 17.8	49.6 ± 16.3	0.613	46.7 ± 17.5	47.2 ± 18.7	0.911	47.3 ± 17.0	43.3 ± 16.4	0.170
GOLD 2017 stages: A/B/C/D,%	28/37/15/20	5/17/0/78	**<0.001**	30/39/15/16	10/26/0/64	**<0.001**	30/42/16/12	12/38/7/43	<**0.001**
*Propensity score matching*	24/36/19/21	0/0/0/100	0.292	25/37/20/18	25/25/0/50	0.132	27/38/22/13	19/34/9/38	**0.021**
LTOT, %	22	65	**<0.001**	19	57	**<0.001**	13	48	<**0.001**
*Propensity score matching*	23	25	>0.999	21	22	>0.999	13	35	**0.002**
mMRC dyspnea grade	2 [1; 3]	3 [2; 4]	**<0.001**	2 [1; 3]	3 [2; 3]	**<0.001**	2 [1; 3]	3 [2; 3]	<**0.001**
*Propensity score matching*	2 [1; 3]	2 [1; 4]	0.864	2 [1; 3]	2 [1.2; 2.7]	0.845	2 [1; 3]	2 [1; 3]	**0.013**
COPD-SS severity questionnaire	13 [8; 18]	19 [16; 22]	**<0.001**	12 [8; 17]	19 [14; 22]	**<0.001**	11 [7.2; 16]	17 [12; 21]	<**0.001**
*Propensity score matching*	14 [9; 18]	15 [8; 19]	0.631	13 [9; 16.7]	15 [10; 20]	0.115	12 [8; 16]	15 [12; 20]	<**0.001**
Charlson index	2 [1; 3]	2 [1; 3]	0.305	2 [1; 3]	2 [1; 4]	0.011	2 [1; 3]	2 [1; 4]	**0.001**
*Propensity score matching*	2 [1; 3]	2 [1; 3.7]	0.960	2 [1; 3]	3.5 [1; 4.2]	0.015	2 [1; 3]	3 [1; 4]	**0.017**
Previous AECOPD	0 [0; 1]	1 [0; 3]	**0.001**	0 [0; 1]	1 [0; 3]	**<0.001**	0 [0; 1]	1 [0; 2]	**<0.001**
*Propensity score matching*	0 [0; 1]	0.5 [0; 1.7]	0.916	0 [0; 1]	1 [0; 1.2]	0.471	0 [0; 1]	1 [0; 1.7]	0.166
Patients with >2 previous AECOPD, %	23	47	**<0.001**	20	44	**<0.001**	16	34	**<0.001**
*Propensity score matching*	23	25	>0.999	21	22	>0.999	17	25	0.278
Previous AECOPD requiring hospitalization	0 [0; 1]	1 [0; 2]	**0.001**	0 [0; 1]	1 [0; 2]	0.155	0 [0; 0]	0 [0; 1]	**<0.001**
*Propensity score matching*	0 [0; 1]	1 [0; 2]	0.779	0 [0; 1]	1 [0; 1]	**0.001**	0 [0; 1]	0 [0; 1]	0.125
Patients with ≥1 previous AECOPD requiring hospitalization, %	30	51	**0.002**	26	54	**<0.001**	24	45	**<0.001**
*Propensity score matching*	31	25	>0.999	28	44	0.144	26	38	0.108

Data are shown as number of patients (percentage), means ± standard deviation or medians [1^st^ quartile; 3^rd^ quartile], unless otherwise stated. Percentages are calculated on non-missing data.

For the full cohort data are calculated on survivors (n = 381, n = 333, and n = 208) and deaths (n = 51, n = 84 and n = 166) in the follow-up of 6-months, 1-year and 3-years, respectively. In the propensity score matching sample data are calculated on survivors (n = 163, n = 144, and n = 97) and deaths (n = 8, n = 18 and n = 52) in the follow-up of 6-months, 1-year and 3-years, respectively.

In full cohort, the percentage of lost in AECOPD without and with BE groups was 4% and 4% (p > 0.999), 8% and 6% (p = 0.591), and 19% and 13% (p = 0.130) in the follow-up of 6-months, 1-year and 3-years, respectively. In the propensity score matching sample, the percentage of lost in AECOPD without and with BE groups was 8% and 4% (p = 0.536), 15% and 7% (p = 0.058), and 21% and 15% (p = 0.336) in the follow-up of 6-months, 1-year and 3-years, respectively.

*Abbreviations:* BMI indicates body mass index; FEV_1_, forced expiratory volume in the 1^st^ second; GOLD, global initiative for chronic obstructive lung disease; LTOT, long-term oxygen therapy; mMRC, modified Medical Research Council; COPD-SS, COPD severity score questionnaire.

## Discussion

The coexistence of both COPD and BE has been recently defined as an emerging phenotype of patients^14^ who experience worse prognosis^15,17,19–21^; however, data on these patients do not consider several baseline covariates as cofounders. Our prospective study, performed for the first time in hospitalized AECOPD patients and using a PSM method, demonstrated that the presence of BE does not worse the clinical impact at admission, the clinical progression, the rate and the risk of short and long-term mortality.

### Prevalence and characteristics associated to BE

Although in literature a large prevalence of BE associated to COPD is reported^4,15–18^, our prevalence in AECOPD patients was slight higher (36% *vs* 27%) in comparison with COPD patients in whom a CT scan was performed to phenotype the heterogeneity of disease^4^. Recent data on distinctive clinical, functional and microbiological phenotypes of patients with BE have shown the prevalence of BE having COPD as the aetiology cause in a percentage of 11%^33^, lower than previous reports (17%)^34^. It is than clear that differences in prevalence of patients having COPD and BE depend on the respective population under consideration.

Baseline characteristics of our patients with BE were consistent with other reports on age^15,17^, low BMI^15–17^, smoking habit^15^, severe obstruction^16,17,22^, greater dyspnoea^16,17^, need for oxygen-therapy^17,22^ and previous exacerbation events^16,17,22^. Concerning our higher prevalence of *Pseudomonas aeruginosa* isolation in patients AECOPD with BE, previous studies on COPD in stable phase confirmed these data^4,16–18,22^. The presence of this pathogen, most frequently in severe patients and during exacerbations^35^, favours the hypothesis that potentially pathogenic microorganisms (PPM) - and *Pseudomonas aeruginosa* is one of the most important PPM - are responsible for the development of BE by an increase in chronic inflammation^36^. Surprisingly, the prevalence of *Haemophilus influenzae* in our cohort with a positive sputum culture (n = 16, 17%) was lower in comparison to AECOPD patients in general^37^, to BE patients^38^, and stable COPD patients^17^. However, this prevalence was similar after PSM (n = 6, 15%) with a similar trend between AECOPD patients with and without BE. A different pathogen detection or a previous use of antibiotics before admission (not collected in this study) may explain the difference in prevalence.

### Clinical impact of BE at admission

To our knowledge, we have reported for the first time data on the impact of BE on clinical presentation of AECOPD patients. In clinical practice, it is common belief that BE patients especially if in association with an AECOPD may have a worse impact. However, our findings demonstrate that clinical and laboratory data of AECOPD with and without BE were similar, except for hypercapnia levels with renal compensation, that appear better in AECOPD with BE, as well the prevalence of ICU admission (Tables 2 and 4). Interestingly, also the early inflammatory profile of AECOPD with and without BE was similar. Although COPD patients may have different profiles in response to pneumonic and nonpneumonic exacerbations^39^, we demonstrated that the presence of BE in AECOPD does not induce a stronger early inflammatory response.

### Mortality related to BE

In patients with AECOPD several predictors of mortality have been identified in a short and long-term period^40^; as well in our data (Table 6), age, BMI, FEV_1_, and LTOT predict the worse prognosis of AECOPD^40^.

There are no published studies evaluating the risk of death based on the presence of BE during an AECOPD, while in COPD patients the association with BE have been reported with^15,17,19,20^ and without^18^ an impact on mortality. However, studies reporting the worse prognosis, also considered for a recent meta-analysis^21^, concern preliminary data with very few enrolled patients^19^ and studies considering patients with evident baseline covariates, including elderly patients^20^, patients with very severe lung functional impairment^19,20^, and patients with chronic respiratory failure needing oxygen-therapy^20^. Moreover, the adjustments in regression analysis leading to more striking estimates supported the hypothesis that confounding cannot account for the result^15^; the role of confounding should always be considered as a possible *alternative storyline*^41^.

### Why a PSM method for our observational data: a comparison with regression adjustment

Historically, regression adjustment has been used more frequently than PS methods to account for differences in measured baseline characteristics between exposed and unexposed subjects. However, there are several reasons for preferring PS-based methods to regression-based methods for reducing the effects of confounding in observational studies.

First, related to the occurrence of BE and baseline covariates to the outcome, it is simpler to determine whether the PS model rather the regression model has been adequately specified. Diagnostics for PS are based on comparing the distribution of measured baseline covariates, between AECOPD with and without BE in the PSM sample. Goodness-of-fit measures in regression models do not provide a test of whether the outcome model has been correctly specified. Furthermore, goodness-of-fit do not allow one to determine the degree to which the fitted regression model has successfully eliminated systematic differences between AECOPD patients with and without BE.

Second, similarly to a randomized controlled trial (RCT), the PS-based methods allow one to separate the design from the analysis of the study, without any reference to the outcome. However, when using regression adjustment, the outcome is always in sight, and the researcher is faced with the subtle temptation to continually modify the regression model until the desired association has been achieved^42^.

Third, there may be increased flexibility when the occurrence of BE is more common than outcome (time-to-event in nature)^43^. When outcome is time-to-event in nature, prior research has suggested that at least 10 events should be observed for every covariate that is entered into a regression model^44,45^. Thus, in some settings, insufficient outcomes may be observed to allow one to adequately adjust for all baseline variables that one would like to include in the regression model.

Fourth, the PS method provides a better assessment of the degree of overlap between the distribution of baseline covariates, comparing the outcome between patients who have a similar distribution of observed baseline covariates. In a setting in which there is a strong separation between the two groups, the analyst may proceed with a regression-based analysis without being aware that the fitted regression model is interpolating between two distinct populations.

In conclusion, PS method allows one to transparently design and analyze our observational study.

### Strength and limitation

The originality of using data about AECOPD patients with BE, the prospective and consecutive nature of the data collection, the large cohort of the patients enrolled, the long-term follow-up, and the statistical method using a PSM are the major strengths of our research. There are however some limitations. First, our study was conducted at a single centre and in only one country; data from international centres are therefore necessary to confirm our findings. Second, we had not chest CT scans for all enrolled patients and we cannot exclude an under estimation of BE. However, in clinical practice at admission to hospital, in an AECOPD patient without a radiological (all patients enrolled had performed a chest X-ray) and a clinical suspicion of BE, the chest CT scan is not performed. We may reasonable hypothesize that really these patients were AECOPD without BE, as we have classified. Moreover, the presence of a chest CT scan has been used as a covariate for the PS model and then all patients considered in PSM cohort (with and without BE) had performed a chest CT scan; this have eliminated the hypothetical bias that patients performing a chest CT scan were worst patients. Finally, the analysis of data excluding patients without a chest CT scan in full cohort (data not shown), after matching of all baseline characteristics produce similar results; however, the total sample size was not adequate to demonstrate the study hypothesis (see statistical analysis). Third, we lack information about the cause of death; in COPD patients, however, the causes of death (respiratory, cardiovascular, others) are not significantly influenced by BE^17^.

In conclusion, our study supports the hypothesis that in AECOPD patients, the clinical impact and prognosis of BE is influenced by several baseline covariates. After matching, with the elimination of confounding, BE does not directly worsen the prognosis of patients in a period until 3-years.
